# Deep learning from multiple experts improves identification of amyloid neuropathologies

**DOI:** 10.1186/s40478-022-01365-0

**Published:** 2022-04-28

**Authors:** Daniel R. Wong, Ziqi Tang, Nicholas C. Mew, Sakshi Das, Justin Athey, Kirsty E. McAleese, Julia K. Kofler, Margaret E. Flanagan, Ewa Borys, Charles L. White, Atul J. Butte, Brittany N. Dugger, Michael J. Keiser

**Affiliations:** 1 Bakar Computational Health Sciences Institute, University of California, San Francisco, CA 94158 USA; 2 Institute for Neurodegenerative Diseases, University of California, San Francisco, CA 94158 USA; 3 Department of Pharmaceutical Chemistry, University of California, San Francisco, CA 94158 USA; 4 Department of Bioengineering and Therapeutic Sciences, University of California, San Francisco, CA 94158 USA; 5 Department of Pediatrics, University of California, San Francisco, CA 94158 USA; 6 Department of Pathology and Laboratory Medicine, School of Medicine, University of California, Davis, Sacramento, CA 95817 USA; 7 Translation and Clinical Research Institute, Newcastle University, Newcastle, UK; 8 Department of Pathology, University of Pittsburgh Medical Center, Pittsburgh, PA 15260 USA; 9 Department of Pathology, Northwestern University, Evanston, IL 60208 USA; 10 Mesulam Center for Cognitive Neurology and Alzheimer’s Disease, Northwestern Medicine, Chicago, IL 60611 USA; 11 Department of Pathology, Loyola University Medical Center, Maywood, IL 60153 USA; 12 Department of Pathology, University of Texas Southwestern Medical Center, Dallas, TX 75390 USA; 13 Center for Data-Driven Insights and Innovation, University of California, Office of the President, Oakland, CA 94607 USA

**Keywords:** Amyloid beta, Histopathology, Deep learning, Consensus, Expert annotators, Algorithms

## Abstract

Pathologists can label pathologies differently, making it challenging to yield consistent assessments in the absence of one ground truth. To address this problem, we present a deep learning (DL) approach that draws on a cohort of experts, weighs each contribution, and is robust to noisy labels. We collected 100,495 annotations on 20,099 candidate amyloid beta neuropathologies (cerebral amyloid angiopathy (CAA), and cored and diffuse plaques) from three institutions, independently annotated by five experts. DL methods trained on a consensus-of-two strategy yielded 12.6–26% improvements by area under the precision recall curve (AUPRC) when compared to those that learned individualized annotations. This strategy surpassed individual-expert models, even when unfairly assessed on benchmarks favoring them. Moreover, ensembling over individual models was robust to hidden random annotators. In blind prospective tests of 52,555 subsequent expert-annotated images, the models labeled pathologies like their human counterparts (consensus model AUPRC = 0.74 cored; 0.69 CAA). This study demonstrates a means to combine multiple ground truths into a common-ground DL model that yields consistent diagnoses informed by multiple and potentially variable expert opinions.

**Supplementary Information:**

The online version contains supplementary material available at 10.1186/s40478-022-01365-0.

## Introduction

There are inherent differences in evaluation within the medical disciplines. This becomes nuanced when differences in classification could lead to high-stakes differences in prognosis, treatment, and prevention measures [1–3]. Pathology in particular can benefit from more standardized approaches that improve quality and reliability of assessments, especially for imaging data [4–10]. However, in cases of discrepancy, it can be difficult to determine what is objective truth [11, 12]. Furthermore, medical professions such as neuropathology are facing dwindling workforces despite high demand for their services [13, 14]. Hence, machine learning algorithms and associated workflows are needed that augment the ability of the expert in addition to taking into account each expert’s ground truth in model development. Here, we present an automated framework to learn from multiple neuropathology experts and provide a robust labeling co-pilot tool that is resilient to discrepancies among different expertises.

Alzheimer’s disease is a neurodegenerative disease where specific proteins accumulate in select neuroanatomic regions [15]. Studying these accumulations and accurately characterizing their presence, localization, and distribution improves understanding of disease pathophysiology [16]. Extracellular accumulations of amyloid beta (Aβ) into plaques are a pathological hallmark of Alzheimer’s disease. Aβ aggregates form diverse plaque morphologies, as well as deposits within blood vessels, termed cerebral amyloid angiopathy (CAA) [17, 18]. These morphologies may change with disease severity and correlate to select clinical features [19, 20]. Historically, the Consortium to Establish a Registry for Alzheimer’s Disease uses a semiquantitative measure of neuropathological phenotypes in its criteria [5]. Having more consistent, quantitative, and precise anatomic measures of Aβ aggregates would standardize research endeavors, provide deeper phenotyping across institutions, and aid in detection of more subtle pathophysiological differences.

Pathological annotation can be a laborious and time-intensive process that leverages the unique training of the expert [7, 16, 21]. In previous work, we automated a single expert’s annotations using DL on images of Alzheimer’s disease pathologies [22]. This approach was later validated by an independent study with a different cohort [23]. However, individual bias of the expert remained, and it was not yet clear if these methods could scale across multiple experts, institutions, and data modalities—all of which are critical for assessing generalizability as well as model robustness [24]. In the current study, we draw on the contributions of five experts across a methodically and geographically diverse dataset.

Even if individual experts could be perfectly augmented by models trained to reflect their annotation expertise, a common ground truth is difficult to ascertain for challenging pathology tasks where experts may have different interpretations. Guan and Hinton [25] trained neural network ensembles to mimic individual doctors, and created a benchmark of ground-truth examples of retinal neuropathy diagnosis by adjudicating decisions among experts. Although an active discussion may be a judicial way to handle more difficult labeling, a consortium of experts is not always available, requires much labor, and presents confounding social factors that may bias labeling. Avoiding human adjudication and performing the task accurately and automatically at scale—while still utilizing diverse expert proficiency—may be optimal and time-efficient as a first-pass or a second-opinion assessment. Other approaches have assigned reliability scores to each expert, and used statistical procedures to estimate ground truth [26–30]. We assessed complementary approaches, by both modeling individual learners and also augmenting a consensus-voting scheme among experts. The consensus models, i.e. wisdom of the expert crowd, learned the final assessment using various voting thresholds, resulting in different levels of sensitivity and precision. The flexibility to choose whether to favor sensitivity or precision in a consensus scheme may be useful depending on the specific labeling task. Furthermore, we hypothesized that learning from a consensus scheme would facilitate learning common signal among experts as opposed to the high individual bias of learning from just a single expert. We evaluated which of these two approaches was the most robust and performant—learning from individual assessments or learning from some consensus of experts. Finally, we built on the approach presented by Guan and Hinton [25], and created ensembles robust to intentionally poor and noisy information.

We evaluated these models in a prospective research study to show the method’s capability to assist pathologists. The DL models accurately adopted annotation patterns of their expert counterparts. We found that models trained from a consensus of experts were able to (1) reproduce consensus annotations, (2) prospectively filter and enrich for neuropathologies in new whole slide images (WSIs), (3) and exceed the performance of models trained from individual experts at the same tasks. We found that modeling a consensus of experts was performant even when evaluated on different benchmarks that were expected to favor individual models, advocating for the robustness of consensus learning. This methodology may be a powerful means to leverage diverse and complementary human expertise—among pathologists and more generally—to create a standardized DL-based co-pilot for pathology assessments free of human adjudication. Furthermore, we present an unprecedented dataset of 150,000 expert-annotated amyloid neuropathologies, specifically collected to support the training of individualized versus cohort-wide CNN models. We are unaware of a dataset to determine neuropathological inter- and intra-rater annotations at this scale. We are certainly unaware of attempts to compare and contrast individualized versus consensus-based CNN models for a pressing clinical problem at this scale. We are releasing the dataset, the neural network code, and the trained CNN model weights openly and without restriction. For us, the most exciting, impactful, and actionable result of the study is that a community consensus approach to deep learning near universally out-performed individualized CNNs trained from individual experts.

## Materials and methods

### Slide preparation

43 WSIs of the temporal cortex were collected from 3 different sites: 17 from the Alzheimer’s Disease research Center at the University of California, Davis, 16 from University of Pittsburgh, and 10 from UT Southwestern. See Additional file 1: Figure S1 and Additional file 2 for associated clinical data. Inclusion criteria provided to each site was stated as follows: “archival Aβ stained slides containing the middle temporal lobe gyrus (other temporal gyri can be within the section as well) from cases having a primary clinicopathological diagnosis of AD and/or minimal AD neuropathologic changes i.e. “normal”) containing some level of amyloid plaques- can be diffuse, cored and/or neuritic (such as CERAD sparse-frequent). Cases containing CAA were also welcome.” Slides were derived from formalin-fixed paraffin-embedded sections and stained with an antibody directed against Aβ. UC Davis used the Aβ 4G8 antibody (BioLegend (formally Covance), San Diego, CA catalog number SIG-39200), University of Pittsburgh used an NAB228 antibody (Cell Signaling Technology, Danvers, MA catalog number 2450), and UT Southwestern used a 6E10 antibody (BioLegend (formally Covance), San Diego, CA catalog number SIG-39320). WSIs were imaged on an Aperio AT2 at 20 × magnification at each of their respective institutions, and digital files were coordinated via a secure Google Shared Drive.

### Data collection and annotation (phase-one)

The study progressed through two phases. We selected 29 of the 43 WSIs at random for phase-one, and used the remaining 14 for phase-two. Both phases had slide representation from each site. We color-normalized the WSIs according to the method presented in Reinhard [31]. Each WSI was uniformly tiled to 1536 × 1536 pixel non-overlapping images. After tiling, we applied a hue saturation value (HSV) filter and smoothing technique to detect candidate plaques, using the python library openCV. We used different HSV ranges for the different stain types as follows: 4G8 HSV = (0, 40), (10, 255), (0, 220); 6E10 HSV = (0, 40), (10, 255), (0, 220); NAB228 HSV = (0, 100), (1, 255), (0, 250).

Each candidate was center cropped to provide a 256 × 256 pixel image. This process yielded 526,531 images for phase-one. We randomly selected 20,099 images from this set to be annotated by our seven annotators. From our previous study [22], we found that model performance began to plateau around 20,000 training examples.

For the first phase of annotation, these 20,099 images were shuffled and placed into a fixed order, then uploaded to an Amazon instance web portal for independent annotation by seven different persons. Five of them were professionally trained experts (E.B., B.N.D., M.E.F., J.K.K., and K.E.M.), while the remaining two were undergraduate novices (J.A. and S.D.) with no formal training in neuropathology. Each person annotated the same 20,099 images in the same exact order in a multi-label classification task using a rapid-keystroke based custom annotation tool (first described in Tang et al. [22], with current code released in https://github.com/keiserlab/consensus-learning-paper). Each 20X image had a bounding box, and the annotators were instructed to label any and all Aβ pathologies found within the box. Annotators had the option to label the pathology as any combination of three classes: cored, diffuse, or CAA. Since WSIs were not specifically selected for CAA, we did not subclassify these pathologies further (i.e. leptomeningeal vs. cortical). Each annotator did have the option to mark an image as “negative,” “flag,” or “not sure.” We did not use these alternative markings for constructing our final image labels (see Additional file 1: Figure S2). Hence, any Aβ class marked as positive was recorded as a positive annotation for that class. Any classes left unmarked were recorded as negative. There were instances in which an annotator marked negative and also marked a positive annotation for any of the three Aβ classes. Whenever this occurred (which was rare, Additional file 1: Figure S2b), we labeled the image as positive for the amyloid pathology indicated.

This process yielded seven different annotation sets (one for each person) for the 20,099 images that were annotated. From the five expert annotated sets, we constructed consensus-of-*n* annotation sets (from *n* = 1 to *n* = 5) for the same 20,099 images. For a given image *i* and Aβ class *c*, the new consensus-of-*n* annotation was recorded as positive if any *n* experts marked image *i* as positive for class *c*, else the image was recorded as negative.

### Training and evaluation of DL models

Of the 29 WSIs for phase-one, we used 20 WSIs for training and 9 separate WSIs for the hold-out test set. The 20,099 phase-one images were divided into a 67% train and 33% hold-out test split. Of the 67% training data, we performed four-fold cross-validation, keeping each fold’s image set consistent across all training and evaluation protocols such that each fold always had a distinct set of images that were not present in any other fold. We chose a four-fold validation to obtain more performance metrics and assess model generalizability across our dataset. We trained one model for each fold of the cross-validation, resulting in four models for each annotation set. Due to the large imbalance between the different Aβ classes, we performed class balancing during training. We calculated the ratios of diffuse to cored (*r1*), and diffuse to CAA (*r2*), and replicated any image with a cored plaque present a total of *r1* times, and any image with a CAA plaque present a total of *r2* times. Models were trained to perform multi-class classification of the three Aβ classes, taking a 256 × 256 pixel image as input and returning three floating point predictions (one for each class) as output.

For selecting hyperparameters, we largely kept the ones from the original study [22] and hence did not perform a large search. We used a four-fold cross-validation to select our hyperparameters, choosing ones that achieved the highest average AUPRC over four validation folds. We performed a sparse search and explored learning rates of 0.001, 0.0002 and weight decays of 0.03, 0.01. Once hyperparameters were set, we trained four CNNs (one CNN for each fold) for each of the twelve annotation sets (seven from our individual annotators, and five from our consensus annotation sets). We trained models for 60 epochs (which in our previous study [22], was enough to allow for model performance to plateau), and saved the best model state that resulted in the highest cored AUPRC performance over the validation set. For an example loss curve from the consensus-of-two model, see Additional file 1: Figure S3. We used an Adam optimizer with a learning rate of 0.001, and a weight decay of 0.03. We used a multi-label soft margin loss as our loss function. During training, the input images were transformed with a random horizontal flip, a random vertical flip, a random 180° rotation, a color jitter, and a random affine transform so that the model would learn to generalize across different inputs. Images were normalized to have zero-mean and unit-variance prior to entering the model.

We converted each image annotation into a floating point representation, such that the binary labels given by the annotators were converted to a more specific continuous value, using information from other annotated bounding boxes that may be present in the image. During the annotation process, each 20× image possibly contained regions that were previously annotated in a different query with a different bounding box. Hence we had instances in which parts of bounding boxes or multiple bounding boxes from different annotations (from the same annotator) were present in a single 20× image. For each image *i*, we used the labeled bounding box information that the annotator provided in order to create a floating point representation for each class *c*. The floating point representation of *c* was the total sum of fractions of positively labeled bounding boxes for class *c* that exists within *i.* An image potentially had more than one positively labeled bounding box within *i,* or many fractions of positively labeled bounding boxes within *i.* Therefore, the floating point representation was possibly greater than 1.0, but must have been greater than or equal to 0.0. Finally, image *i* was considered positive for class *c* if the floating point representation of class *c* was greater than 0.99. This final binary label was used for training and evaluation.

During evaluation of a model, no transforms were applied except for normalization. For each type of model, we evaluated our models by taking each model from each fold and evaluating it on the hold-out test set. The reported metrics were the average over these four evaluations. For example, the reported phase-one results for the consensus-of-two model was the average performance of all four consensus-of-two models, one for each of the four cross-validation folds, each evaluated on the hold-out test set. The full source code used for training and evaluation can be found at https://github.com/keiserlab/consensus-learning-paper. We used the PyTorch library [32] for all training and evaluation.

The CNN architecture was a simple repeated motif of 2D convolution with a 3 × 3 filter and padding of size one, batch-norm, ReLU activation, and 2 × 2 max pooling with a stride step of size two. We started with sixteen filters, and with each application of the motif, we increased the number of filters by sixteen until we had 96 filters. We then applied a final affine layer to obtain our three class scores.

### Assessing consensus model and benchmark superiority

When assessing the consensus models versus the individual-expert models, we applied four different benchmark schemes using only the hold-out test set annotations, and calculated the average performance of the consensus models minus the average performance of the five individual-expert models. “Self” means that each model was evaluated by its own benchmark (i.e. a model trained under A’s annotation set is evaluated according to the annotations in A’s hold-out test set). For instance, a consensus-of-two model with benchmark “Self” was evaluated according to how well the predictions matched with the hold-out test labels provided by the consensus-of-two annotations. This was done for each of the four consensus-of-two models (one for each fold), and averaged. “Consensus benchmarks” means that all models were evaluated on how well their predictions matched on average with each of the five consensus benchmarks (consensus-of-*n* from *n* = 1 to *n* = 5). We averaged performance across these five consensus benchmarks. “Individual benchmarks” means that all models were evaluated on how well their predictions matched on average with each expert benchmark. We averaged performance across these five expert benchmarks. “All benchmarks” is evaluating a model across the five consensus benchmarks and five expert benchmarks, and then averaging these results. For the consensus-of-two superiority results, we performed the same procedure, except only the consensus-of-two model and consensus-of-two benchmark were evaluated to represent the consensus strategy.

We used a two-sample, one-sided Z-test to assess statistical significance. The null hypothesis was that the average of the consensus performance and the average of the individual experts’ performance were the same. The alternative hypothesis was that the average of the consensus performance was greater than the average of the individual experts’ performance. *P*-values were reported using a standard lookup table. The sample sizes for each of the two samples (consensus and experts) are as follows: 20 for “self”; 100 for “consensus benchmarks”; 100 for “individual benchmarks”; and 200 for “all benchmarks”. Each sample was a model’s performance on a specific benchmark.

### Model interpretability

We used guided Grad-CAM to obtain the saliency maps from the different models (https://github.com/utkuozbulak/pytorch-cnn-visualizations). For analyses requiring image binarization, we chose incremental pixel thresholds such that at threshold *t*, any signal less than *t* is assigned 0 value, while anything greater than or equal to *t* is assigned a maximal pixel value of 255. To calculate the structural similarity index measure (SSIM) between the novice CAM and the consensus-of-two CAM, we used a standard SSIM function from the skimage library.

For analyzing the CAM patterns of the novice CAMs versus the consensus-of-two CAMs, we constructed a subtraction map of the binary consensus CAM minus the binary novice CAM for each 256 × 256 pixel image. Each pixel of this map is classified as one of three classes: signal (ON) in the novice CAM and no signal (OFF) in the consensus CAM, or no signal in the novice CAM and signal in the consensus CAM, or a match between the two. For the CAM subset analysis, we calculated both the total fraction of the binary consensus CAM that was activated in the binary novice CAM at the same corresponding pixel locations, and also the total fraction of the novice CAM that was activated in the consensus CAM at the same corresponding pixel locations for each pixel threshold.

### Ensemble training and evaluation

For each of the four folds of the cross-validation, we linked each of the five individual-expert CNNs trained from that fold with a sparse affine layer. The individual-expert CNNs were frozen such that no backpropagation occurred in the individual-expert CNNs. The only weights that were updated were the sparse affine weights, which weight each individual-expert CNN’s final class output.

For each annotation set (five expert sets, and five consensus annotation sets), four ensembles (one for each cross-validation) were trained using the same training data that their constituent CNNs used for training. Holistically, having four cross-validation folds, five experts, and five consensus schemes, resulted in 40 ensemble models, each independently trained to reproduce a specific annotation set. Ensemble training occurred for 60 epochs, with the same hyperparameters that were used to train the single CNNs.

After training, each ensemble model was evaluated on the images from the hold-out test set, and on every benchmark (five expert benchmarks, and five consensus benchmarks). We aggregated ensembles that were trained to mirror the same annotation set but belonged to different cross-validation folds. Hence, every permutation of (1) ensemble model (aggregated across cross-validation folds) and (2) benchmark was assigned an average AUPRC score. This resulted in 100 average AUPRC scores (10 aggregated ensembles, 10 hold-out test benchmarks). To assess ensemble superiority over single CNNs for a given benchmark, these 100 average AUPRC scores were compared to the 100 average AUPRC scores of the single (non-ensemble) CNNs. This resulted in 100 comparisons of ensemble models versus single CNNs, with both model types evaluated on equivalent benchmarks and an equivalent image set.

For ensembles that contained a random labeler CNN, we trained a separate CNN on a random annotation set that kept the same class ratios of cored, diffuse, and CAA as the ones present among the five expert annotations. This ratio was determined by averaging the class ratios of each of the five expert annotation sets. We then linked this CNN trained on random labels with the five expert CNNs using a learnable sparse affine layer. Likewise, for ensembles with five random labeler CNNs, we trained five independent CNNs on five different permutations of the randomly labeled annotation set. We then linked these five random labeler CNNs with the five expert CNNs using a sparse affine layer. For both the ensemble with a single random labeler, and the ensembles with multiple random labelers, we used the same training procedure as the normal ensembles (i.e. ensembles without any random labeler present).

In evaluating the performance of ensembles with any number of random labeler(s) present, we performed the same evaluation procedure as for the normal ensembles. For comparing performance between ensembles with any random labeler versus performance of normal ensembles, we compared the final average AUPRC values for these two model types and for each of the ten benchmarks. This resulted in 100 comparisons between normal ensembles and ensembles with a random labeler, and 100 comparisons between normal ensembles and ensembles with five random labelers. In every comparison, everything was kept constant except for the choice of model architecture.

### Setup of phase-two prospective validation and analysis

To assess use in a real-world clinical research setting, we designed a second, prospective, stage of the study (phase-two) to assess whether models could usefully serve as a prospective filter by identifying comparatively infrequent cored plaques and CAAs for new patients. Whereas cored plaques and CAAs have low prevalence (12% and 2% in phase-one, respectively), we posited that models from phase-one could filter a large and previously unseen dataset of primarily diffuse plaques to enrich for these rare but important neuropathologies. We hypothesized these models could prospectively predict what their annotators were going to label. Moreover, given our initial findings that a consensus-of-two model was more effective and robust than individual-expert models, we compared their prospective capabilities. Annotators were told only that the study would proceed in two phases, but were given no information regarding data selection.

All annotations were performed on the same web interface and by the same expert and novice annotators from phase-one. Each annotator received a total of 10,511 images to label spanning four different categories of images: self-repeat, consensus-repeat, self-enrichment, and consensus-enrichment. The same ordering of these four image categories was given to each annotator for consistency. The exact images given to annotators differed only in the self-repeat and self-enrichment categories. Self-repeats and consensus-repeats were images previously annotated during phase-one, and were collected to assess how consistently annotators were able to label images. By contrast, self-enrichment and consensus-enrichment images came from 14 WSIs spanning 14 new patients that were completely separate from the 29 WSIs from phase-one.

For the self-repeat images, which are simply repeats of a subset of images the annotators already saw in phase-one, we first selected a total of 600 images. We first selected all of the images that were marked as positive for CAA. CAA was rarely annotated during phase-one, and we wanted to include all of them. Once all of the CAAs were included, we selected a random subset of images that were marked as positive for cored until we had 400 images. If there weren’t enough cored positive images to make up 400, then we took as many as possible. For the remaining images we randomly selected diffuse positive images to make up a total of 600 images. We enforced having no duplicates. We then triplicated and randomly rotated these 600 images by 90° increments, and shuffled the resulting 1800 images. This entire procedure of obtaining the self-enrichment image set was done independently for each expert and each undergraduate novice, resulting in different sets of self-repeat images given to different annotators.

Likewise for the consensus-repeat set, we took images exclusively from the set of phase-one images that the annotators already labeled. For each of the three Aβ classes, we randomly selected 250 images that were positive for this class according to a consensus-of-two strategy. From this list of images, we removed duplicates, which occurred because some images were positive for multiple Aβ classes according to a consensus-of-two. This resulted in a total of 745 images, which were then triplicated and randomly shuffled, resulting in our final consensus-repeat set of 2235 images. This set was given identically to all of the annotators during phase-two.

For the self-enrichment images assigned to annotator A, we used the individual-expert CNN trained on A’s phase-one annotations to enrich for images that the model predicted as having an important but minority plaque present (cored or CAA). We chose to use the models from fold three of the cross-validation for enrichment because this yielded the greatest performance over the validation set. All images came from a hold-out set of images that the annotators did not see during phase-one of annotation. This hold-out set consisted of 275,880 images total. From this hold-out set, we randomly selected 800 images with a model prediction threshold > 0.90 for the cored class. Next, the 275,880 images were sorted according to the model’s CAA prediction confidence, and the top 800 images with highest CAA confidence were included. After collecting this set of 1600 images, we included all of the image neighbors that had at least a 20% bounding box overlap of a plaque with any of these 1600 images. Afterwards, we randomly shuffled this resulting list, and took a random subset of 3000 images to use for our final self-enrichment set. This procedure was repeated for each of our seven human annotators. We calculated the overall intra-rater agreement for each annotator by averaging the accuracy of how consistent each annotator was over each set of replicated images. Each replicated set had four total images (one annotation from phase-one, and three annotations from phase-two).

For consensus-enrichment images, we used predictions from the consensus-of-two model to enrich for images. We randomly pulled 750 images that the consensus-of-two model predicted as positive for cored, and an additional 750 images that the same model predicted as positive for CAA. From this resulting set of 1500 images, we found their image neighbors that had at least a 20% bounding box overlap of a plaque with any of the 1500 images. We randomly selected from these neighbors until we had a total of 3476 images for the final consensus-enrichment set. If an image achieved high rank by both self-enrichment and consensus-enrichment, it was assigned with equal probability to either self or consensus.

These four image sets were given to the annotators, such that the ordering of the image category was randomly shuffled, but fixed and identical among each annotator. Upon completion of annotation, we analyzed the model’s prospective performance to match annotations given during phase-two. We stipulated two different benchmarks that could be derived from these new annotations: the individual-expert benchmark, which simply assigned what the individual annotator labeled as the truth labels, and the consensus benchmark, which used the consensus-of-two strategy to assign truth labels. We used the individual CNN models to make class predictions for the 10,511 images of phase-two, independent of the human annotators and their labels. We did the same for the consensus-of-two model, and made class predictions for each of the 10,511 images. Model predictions were completely hidden from the annotators, as well as any experimental details of how the images were selected. For phase-two analysis, we define an individual-expert model as a model trained on one of the expert’s annotation sets from phase-one, and a consensus model as the model trained on the consensus-of-two annotation set from phase-one. All label data used to assess model performance during phase-two came from this second phase of annotation.

To assess performance of individual-expert models under the individual benchmark, we compared the predictions of each individual-expert model trained on annotator A with the labels that annotator A gave during phase-two (undergraduate novices were excluded from this analysis). This was repeated for each expert annotator model and the five results were averaged. To assess performance of individual-expert models under a consensus-of-two benchmark, we compared the individual-expert model’s predictions with the labels provided by a consensus-of-two scheme. This was done for each expert annotator model and the five results were averaged. For the consensus model and individual benchmark case, we compared the consensus model’s predictions with the labels provided by annotator A*.* This was repeated for each of the five professional annotator labels, and these five results were averaged. For the consensus model and consensus benchmark case, we compared the consensus model’s predictions with the label set provided by a consensus-of-two strategy. There was only one consensus-of-two model and one consensus benchmark, resulting in no variability and no averaging.

## Results

### We curated a multi-annotator dataset and found annotation differences among experts and consensus schemes

In prior work [22], we developed a convolutional neural network (CNN) pipeline to automatically identify three different Aβ neuropathologies for a single expert. In this current study, we generalized this method to five experts (NP1-NP5) and two novice annotators (UG1 and UG2). Oftentimes, pragmatic DL applications are trained on data with limited diversity, resulting in inability to effectively generalize to data outside their training corpus [33]. To counter this, we validated our method using a dataset of 43 WSIs obtained from three different institutions, where each used different histological staining procedures (Methods). Additional file 1: Figure S1 contains demographic information on these 43 research patients.

**Fig. 1:**
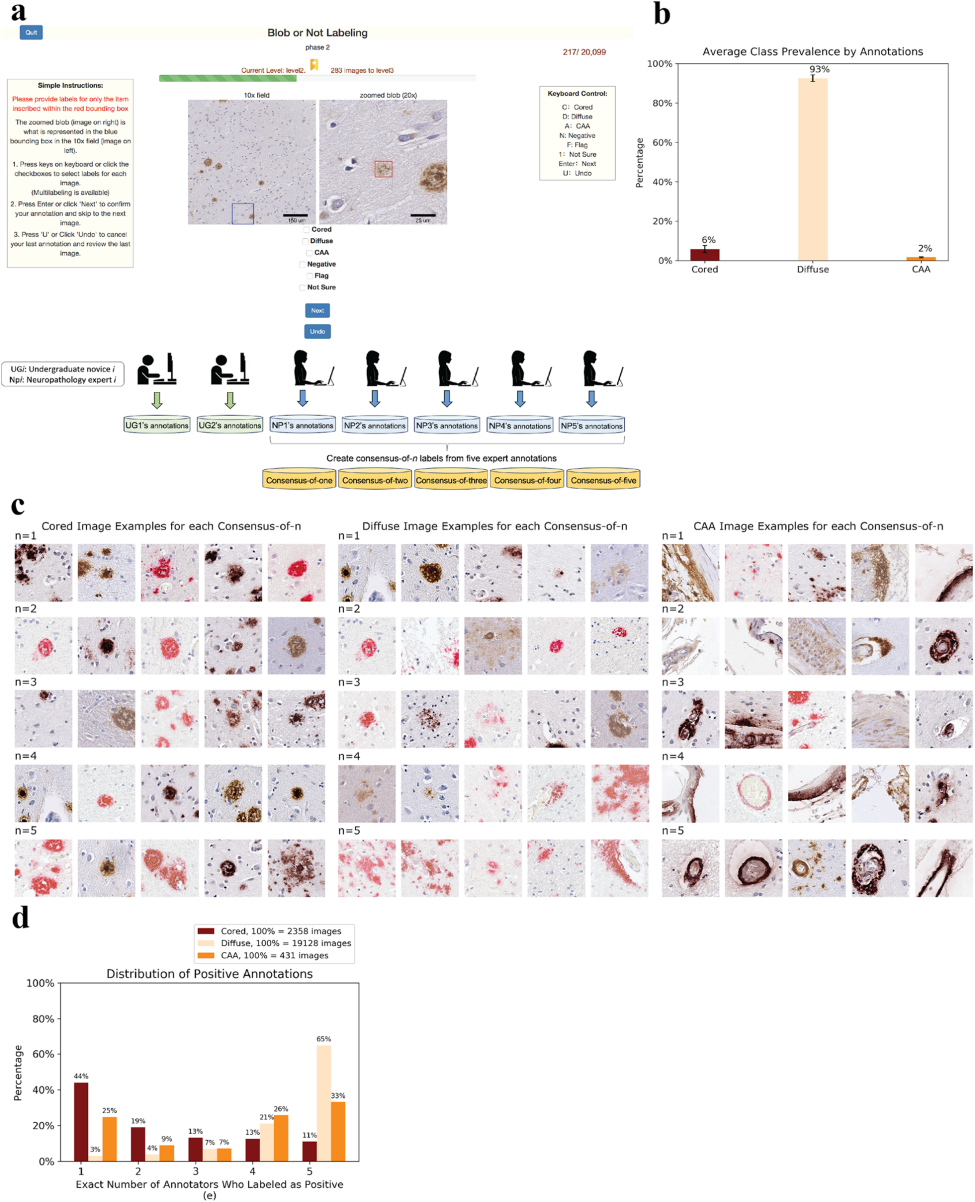
We curated annotations of Aβ neuropathologies from multiple experts, and found differing degrees of consensus. **a** Five experts (NP) and two undergraduate novices (UG) used a custom web portal for annotation. Each annotator labeled the same set of images in the same order. From the expert annotations, we constructed consensus-of-*n* labels (*n* = 1 to *n* = 5) for the same 20,099 images. **b** Average class distributions are consistent across the seven annotators. The y-axis plots average frequency, while the x-axis plots the Aβ class. **c** Representative images illustrating consensus-of-*n* strategies applied to each Aβ class, with rows progressing from top to bottom in order of increasing consensus. For a consensus-of-*n* image, at least *n* experts labeled the image as positive for the designated class*.* Each image was randomly and independently chosen from the set of images. **d** Positive annotation distributions differ by Aβ class. The x-axis plots the exact (not cumulative) number of annotators who gave a positive label. Hence, when *e* = 1 and *e* = 5 this is equivalent to a consensus-of-one and consensus-of-five respectively. For *e* = 2, 3, or 4, this is not equivalent to an at-least-*n* consensus strategy. The y-axis plots the frequency. Each class has a different count of total positive labels (indicated in the legend). This total count represents the total number of images with at least one expert identifying the class. Each image may have multiple classes present

We organized the study into two phases of data collection. In phase-one, we collected independent annotations from the seven annotators on the same 20,099 images derived from 29 WSIs. As in previous work, we color-normalized the WSIs [31], identified candidate Aβ aggregates with conventional computer vision techniques, and center-cropped these candidates to provide 256 × 256 pixel images for final annotation (Methods). We arranged these candidates in a randomly shuffled but fixed ordering, and uploaded them to a custom web interface for expert annotation (Fig. 1a). We recruited five neuropathology experts and two undergraduate novices from five different medical institutions to annotate the images. Annotators performed a multi-labeling task and independently labeled an image as any combination of “cored,” “diffuse,” and “CAA.” The annotators also had options of marking “negative,” “flag,” and “not sure,” but we did not use these to alter our image labels (Additional file 1: Figure S2). As in previous work, the diffuse class was most prevalent (Fig. 1b).

To draw on the complementary proficiencies of multiple experts, we introduced the concept of a “consensus-of-*n*” strategy to label pathologies. In the consensus-of-*n* strategy, an image was labeled positive for plaque *p* if at least *n* experts positively labeled the image as presenting *p*. Otherwise, the image was assigned as negative for *p*. A consensus-of-one was the most permissive strategy, in which only one expert needed to positively identify *p* for this image to be labeled as positive for *p*. A consensus-of-one (logical set union) maximizes sensitivity, while a stricter consensus-of-five (logical set intersection) maximizes precision. We created five different annotation datasets by applying the consensus-of-*n* strategy to the experts’ annotations, from *n* = 1 to *n* = 5. Each consensus annotation dataset combined information from all five experts’ annotations using this thresholding scheme, and consisted of labels for the same set of independently annotated 20,099 images. Images for each Aβ class and each consensus strategy are shown in Fig. 1c.

Qualitatively, we saw more phenotypic uniformity as more agreement was reached from *n* = 1 to *n* = 5. This held especially for the diffuse class in which some *n* = 1 images resembled the phenotype for cored plaques, but as we increased to *n* = 5, the classical phenotype emerged of sparse and scattered Aβ protein. A complete consensus-of-five experts was reached for 65% of images labeled as a diffuse plaque from any expert (Fig. 1d). For the CAA class, the classical ring structure around blood vessels emerged as *n* increased. Complete consensus-of-five was reached for 33% of images with any CAA annotation (Fig. 1d). We did not specify CAA subtypes [34, 35]. For the cored class however, there was no smooth qualitative progression, visually indicating more differences and idiosyncrasies in identification. Complete agreement on a positive label only occured for 11% of cored-plaque labeled images. There were many cases in which only one annotator identified a particular cored plaque, with this consensus-of-one scenario making up the majority (44%) of cored-plaque images (Fig. 1d).

**Fig. 2:**
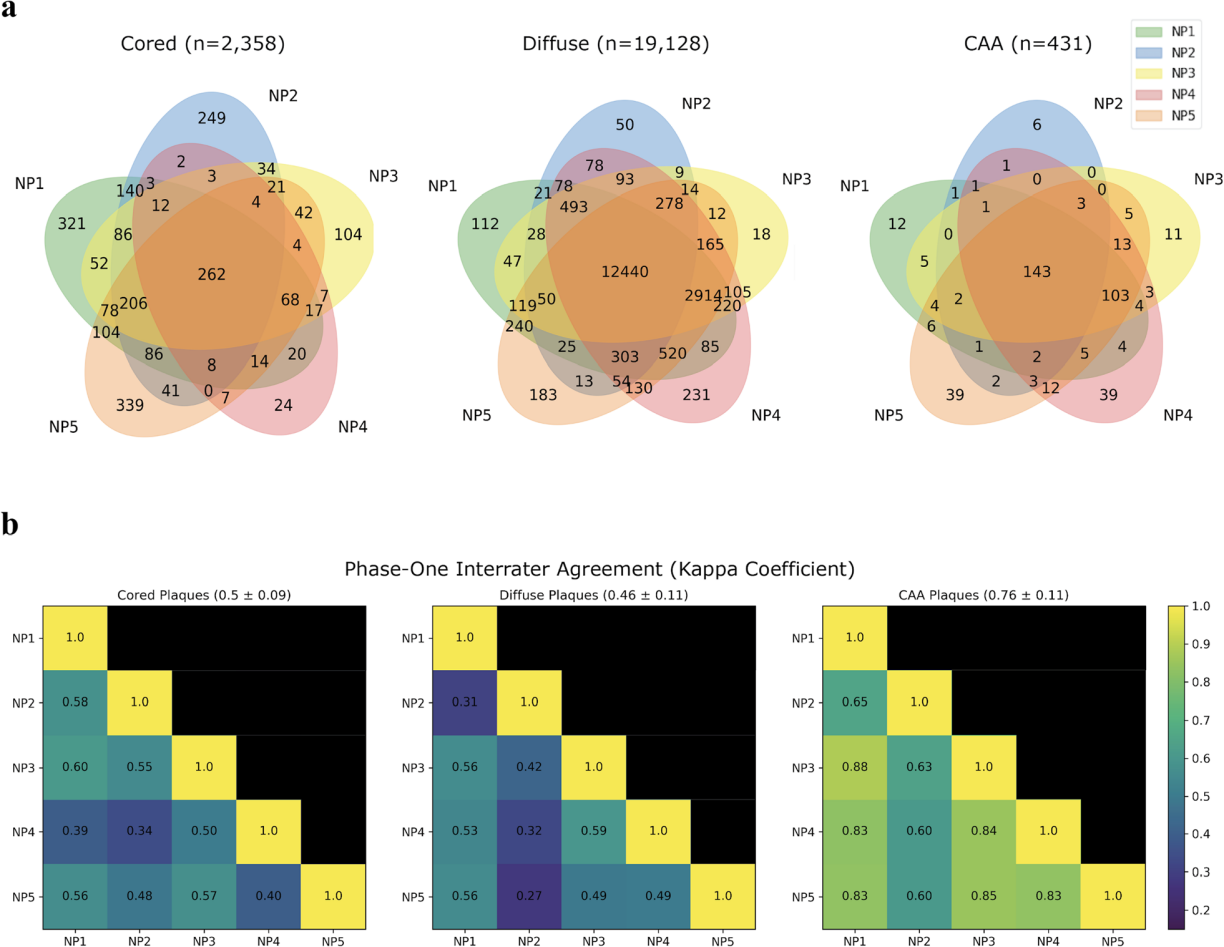
Inter-rater agreement varies by class and annotator. **a** Venn diagrams by class, with overlaps of each permutation of NP1 through NP5. Each overlap shows the count of how many images are all positively annotated by the experts included in that overlap. Areas are not to scale. **b** Kappa coefficients [36] indicating agreement between each pair of experts. A high kappa coefficient indicates high inter-rater agreement between two annotators, with kappa = 1.0 indicating perfect agreement, and kappa = 0.0 indicating no agreement other than what would be expected by random chance

To assess patterns of inter-rater agreement, we calculated the Cohen’s kappa coefficient [36] between every pair of experts (Fig. 2). There was low average agreement for the diffuse class (kappa = 0.46 ± 0.11) despite the fact that a complete consensus (*n* = 5) for it was the most common case out of any consensus. Hence, for diffuse cases without complete agreement, experts varied greatly. For CAA cases, average inter-rater agreement was much higher (kappa = 0.76 ± 0.10). Agreement for the cored cases was low (kappa = 0.50 ± 0.08). No single expert was a clear outlier in annotation across all Aβ classes. NP2 differed most with the other annotators for the diffuse and CAA classes, and NP4 differed most for the cored class.

### CNNs mimic human annotators, and consensus CNNs mimic a consensus of experts

CNNs are a powerful class of DL networks particularly useful for analyzing image data [37–39]. We found CNNs accurately learned the specific annotation behavior of both humans and consensus strategies. Using the five expert annotation datasets, we trained CNNs to reproduce each human’s annotations. We also trained independent CNNs to reproduce each consensus-of-*n* strategy.

**Fig. 3:**
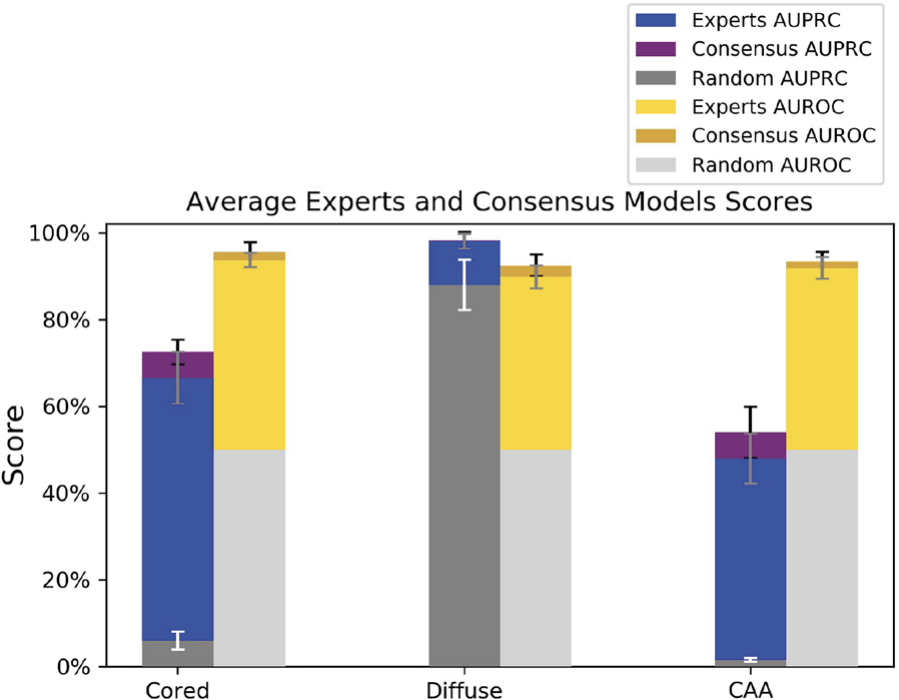
We trained models to learn human annotation behavior and consensus strategies. Consensus models matched or outperformed individual-expert models in average AUROC and AUPRC, per stacked bar graphs. Error bars show one standard deviation in each direction. The y-axis indicates the score on the hold-out test set for each Aβ class (x-axis). No novice models were included in this evaluation. For the AUPRC metric, the consensus model achieved 0.73 ± 0.03 for cored, 0.98 ± 0.02 for diffuse, and 0.54 ± 0.06 for CAA. The individual-expert models achieved 0.67 ± 0.06 for cored, 0.98 ± 0.02 for diffuse, and 0.48 ± 0.06 for CAA. Random baseline performance for AUPRC is the average prevalence of positive examples. Average random baselines for individuals-experts were equivalent to those of consensus strategies (variance of individual-experts shown): 0.06 ± 0.02 for cored, 0.88 ± 0.06, and 0.02 ± 0.004 for CAA. For the AUROC metric, the consensus models achieved 0.96 ± 0.02 for cored, 0.92 ± 0.02 for diffuse, and 0.93 ± 0.02 for CAA. The individual-expert models achieved 0.94 ± 0.02 for cored, 0.90 ± 0.03 for diffuse, and 0.92 ± 0.03 for CAA. All models were evaluated on their own benchmark (i.e. a consensus model was evaluated on its respective consensus benchmark, and an individual-expert model was evaluated on its expert’s benchmark)

We randomly assigned each WSI to either the training set or test set. Of the 20,099 center-cropped images, we held out 33% as a test set, and the remaining training images were split into a fourfold cross-validation (Methods). Both the models trained on individual-expert annotations and the models trained on the consensus annotations generalized and performed well on the hold-out test set (Fig. 3); they had high area under the receiver operating characteristic (AUROC) and area under the precision recall curve (AUPRC). We evaluated each model according to the labels of the annotation dataset on which it was trained. The consensus models mimicked consensus strategies, and the individual-expert models mimicked their corresponding expert annotators. Interestingly, the individual-expert models captured their human inter-rater agreement patterns, further suggesting that they were mimicking their human counterparts (Additional file 1: Figure S4). For every scoring metric and Aβ class, consensus models performed slightly better than individual-expert models (Fig. 3). On average, they were able to more accurately reproduce the consensus strategies than the individual-expert models were able to reproduce their specific human annotators. There were substantial performance differences among the Aβ classes. All models were able to accurately reproduce the annotations of the diffuse class, which was far more ubiquitous than cored and CAA. For these crucial but minority classes, AUROC and AUPRC performance was lower likely due to less available training examples (e.g., 1–2% for CAA class prevalence, depending on label strategy). Performance by stain was largely consistent, except for the CAA class with 6E10 staining, which had lower and more variable performance (Additional file 1: Figure S5). Color-normalization had little effect on performance, except for the CAA class (Additional file 1: Figure S6).

### Consensus provided superior models and annotation benchmarks

**Fig. 4:**
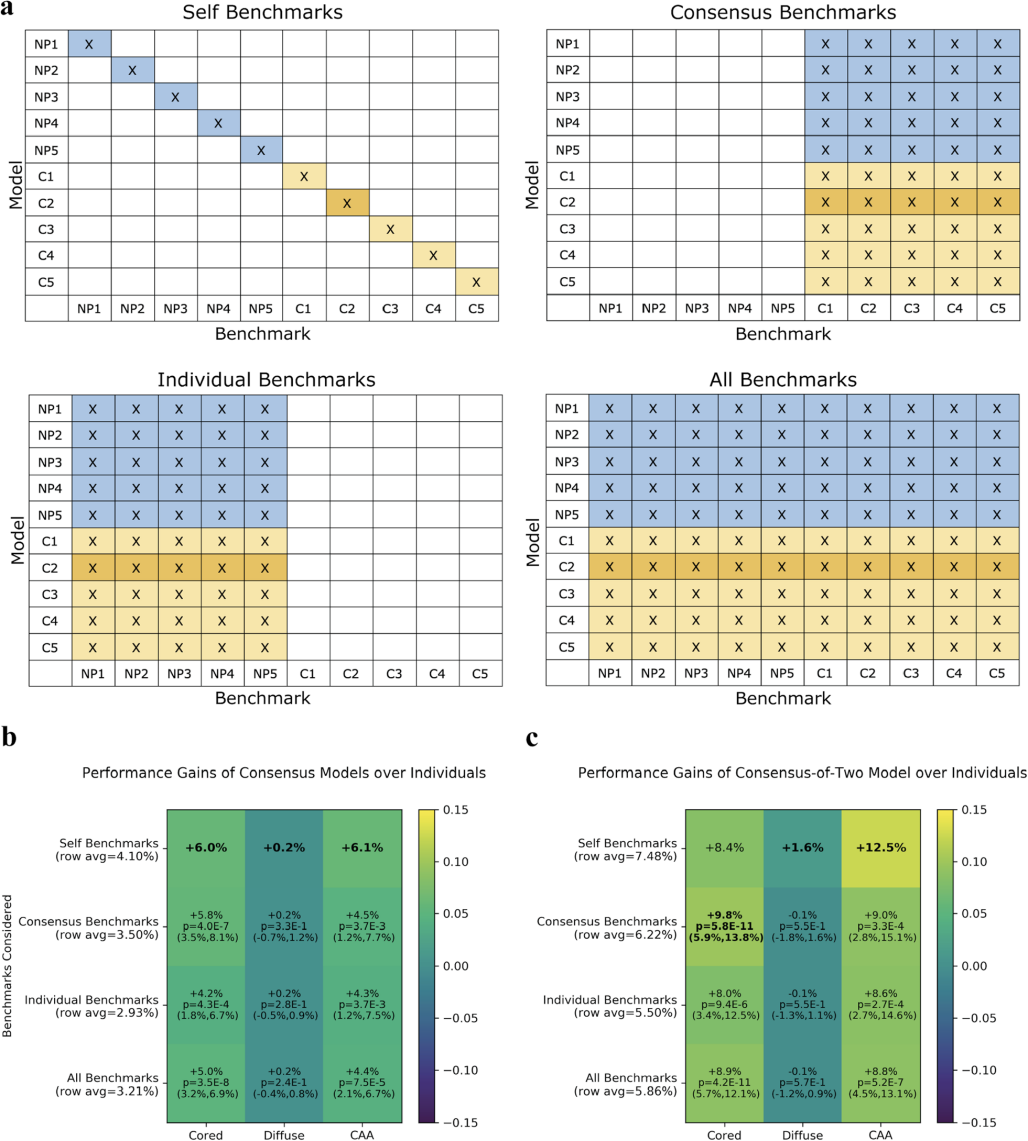
Consensus models performed better than individual-expert models across all benchmarks. **a** Four evaluation benchmark schemes to compare consensus models with individual-expert models. The row indicates the model and the column indicates the benchmark. For each evaluation scheme, the average AUPRC of the blue region (individual-expert models) is compared with the average AUPRC of the gold region (consensus models) over the hold-out test set. The consensus-of-two is dark-gold for emphasis. The “self benchmarks” scheme was the most internally-consistent scheme that evaluated each individual-expert model according to the labels of its annotator (i.e. its own benchmark). For consensus models, the self benchmark corresponded to labels derived from the matching consensus-of-*n* strategy. The “consensus benchmarks” scheme independently evaluated each model on every consensus-of-*n* annotation set from *n* = 1 to *n* = 5. The “individual benchmarks” scheme independently evaluated each model on each of the five individual-expert benchmarks. The “all benchmarks” scheme evaluated each model on its average performance across all benchmarks. **b** Performance gains of consensus models over individual-expert models. Values are reported as the absolute AUPRC difference. We calculated *p*-values of the comparisons using a two-sample Z-test (Methods). *P*-values for the self-benchmark are not included because the sample size (*n* = 20 comparisons) is not large enough to assign significance. 95% confidence intervals shown in parentheses. The row indicates the type of benchmark considered when evaluating the model performance differentials, while the column shows the Aβ class being evaluated. Highest performance differential for each Aβ class in bold. **c** Heatmap as in **b**, for only the consensus-of-two model versus the individual-expert models. For this consensus-of-two model evaluation, only dark-gold regions in **a** corresponding to the consensus-of-two model are compared to the blue region

We found that learning from a consensus strategy resulted in consistently higher model performance than learning from individual-expert annotations. Since there was no clear best or established evaluation benchmark strategy across the test-set images (with each image having five independent expert annotations), we compared the consensus models with the individual-expert models using four different benchmark schemes (Fig. 4a).

For all four benchmark schemes, the consensus models had superior average AUPRC performance versus individual-expert models (Fig. 4b). We found this surprising, especially for the “individual benchmarks” case in which all models were evaluated with the different experts’ annotation benchmarks. We had expected individual-expert models to perform the best on this benchmark, because they were trained specifically to mimic these same annotators. Instead, we observed the consensus models—which in this scenario were trained under a different annotation dataset than the one that they were being evaluated with—consistently performed better on average than the individual-expert models across all Aβ classes (Fig. 4b). Of the five consensus schemes, the consensus-of-two model achieved the highest average AUPRC (0.70 ± 0.24) and AUROC (0.88 ± 0.13) when evaluated against all benchmarks and all Aβ classes. On average, this consensus-of-two model substantially outperformed individual-expert models for all benchmark schemes and for all Aβ classes, except for the diffuse class in which performance was roughly equivalent (Fig. 4c).

When we did the converse and compared annotation *benchmarks* as opposed to comparing *models*, we observed annotation datasets from consensus-of-*n* strategies provided a more robust benchmark yielding greater apparent average model performance, regardless of which models we evaluated (Additional file 1: Figure S7a, b). Similarly, regardless of training strategy, models performed better on the consensus-of-two benchmark than on individual-expert benchmarks, for all Aβ classes (Additional file 1: Figure S7c).

### Model interpretation reflected differences in human expertise

**Fig. 5:**
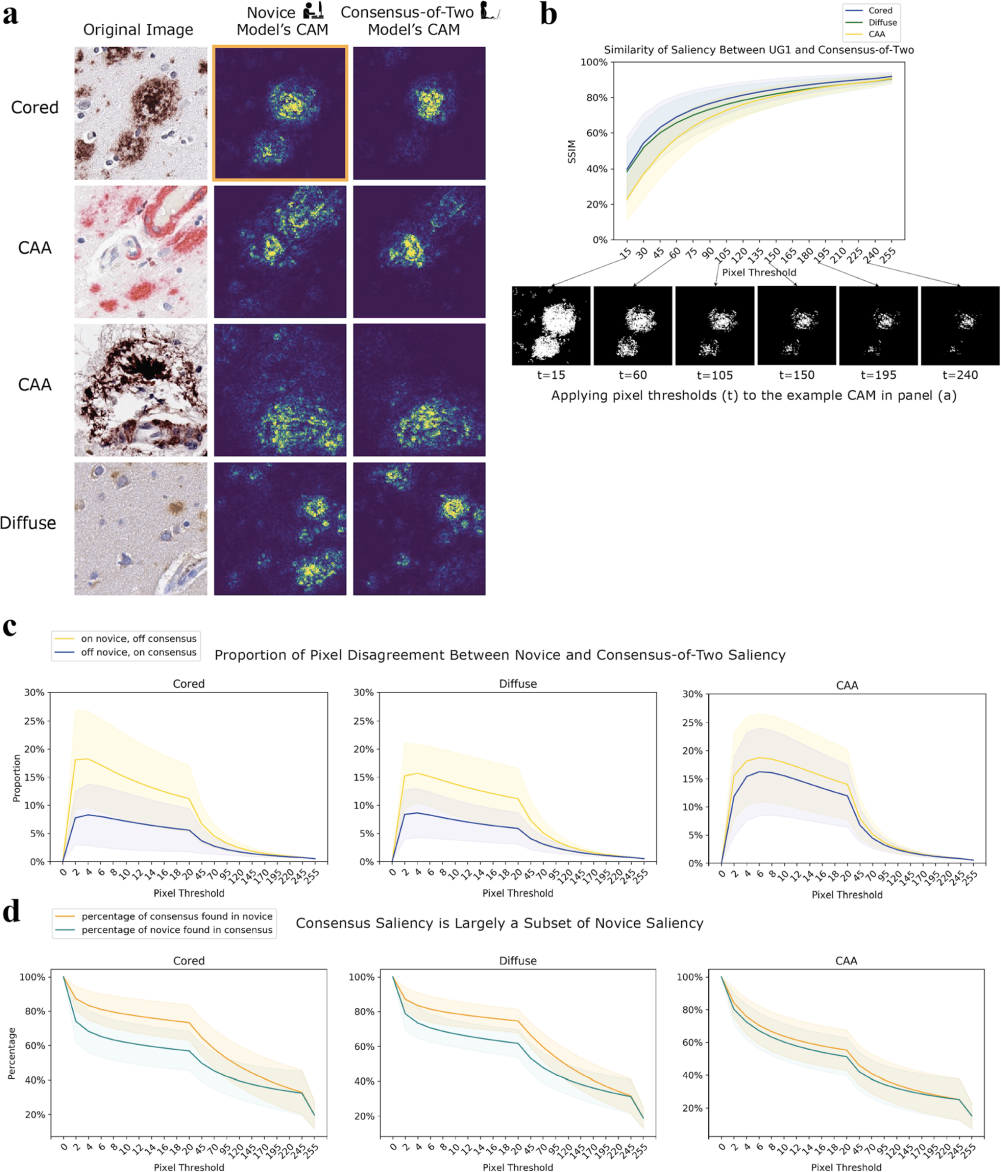
Class activation maps (CAM) of DL models indicate progression of human expertise. **a** Novice CAMs are more diffuse than expert CAMs. The original image (leftmost column), the CAM of the novice model trained on UG1’s annotations (middle column), and the CAM of the consensus-of-two model (rightmost column). CAMs are plotted with a false-color map such that bright regions correspond to high intensity regions with high salience. **b** Although expert and novice CAMs differ, they converge on the same pixels. We progressively assess the structural similarity index (SSIM) [44] between novice CAMs and consensus-of-two CAMs across the entire test set of images. The CAMs show the most similar salience by SSIM (y-axis) at the highest pixel thresholds as we increment the threshold (x-axis) used to binarize the images before comparison. Binarized examples are shown of one CAM from **a** (boxed in orange). **c** Comparing the novice CAMs and the consensus-of-two CAMs, we classify each pixel location into two categories: ON in the novice CAM and OFF in the corresponding consensus CAM (yellow), or OFF in the novice CAM and ON in the consensus CAM (blue). ON and OFF are determined by binarizing the images at pixel threshold *t* (x-axis). Y-axis shows the proportions at which these two cases occur. Zoomed inset highlights disagreement between CAMs. **d** Consensus CAM pixels are mostly contained within the novice CAM. The x-axis plots the varying pixel thresholds, while the y-axis plots the percent overlap of either how much of the consensus CAM pixels are a subset of the novice CAM (orange) or how much those of the novice CAM are a subset of the consensus (cyan)

Models learned human-interpretable, pathologically-relevant, and granular visual features for each Aβ class. By incorporating saliency mapping methods [40, 41] to interpret the model’s rationale, we found models focused on pixels corresponding to boundaries of Aβ pathologies and excluding boundaries of extraneous deposits in a way that was task (i.e., pathology) specific—even though this granularity of information was not provided during training (Fig. 5a).

To investigate the impact of field expertise, we also trained models from the annotations of two undergraduate novices. We observed that a model trained on a consensus of two experts had more focused and specific feature saliency than a model trained on an individual undergraduate novice. To visualize what parts of an image were important to a model’s decision making, we used guided gradient-weighted class activation mapping, or “guided Grad-CAM” [40] (Methods). When we compared class activation maps (CAMs) of the two models—consensus-of-two versus an undergraduate novice—we observed the CAMs of the novice’s model were more diffuse than CAMs of the consensus-of-two model (Fig. 5a). Qualitatively, the individual novice models attributed greater salience to pixel features that were not important to the classification task, while the consensus-of-two model focused on relevant morphologies. Therefore, the model trained on consensus across greater expertise appeared to be a more specific feature detector. These results held for both novice annotators (Additional file 1: Figure S8). However, subsequent studies with additional novices are needed to investigate this trend. The consensus CAMs also seemed more specific than the individual-expert CAMs (Additional file 1: Figure S9).

Quantitatively, for each Aβ class and for each image in our test set, we compared its novice CAMs to its consensus-of-two CAMs. For each pair of CAMs derived from the same image and same class, but different model, we binarized the CAMs across incrementing thresholds (Fig. 5b). The majority of activations matched between the novice and consensus-of-two model at the different thresholds. In regions where they did not match, the novice CAMs tended to have signal while the consensus-of-two CAM did not (Fig. 5c). Figure 5b shows binarized image examples at different thresholds. At high pixel thresholds the images became more similar as the granular features of the image were lost. Furthermore, we computed the fraction of the consensus-of-two CAMs found within the novice CAMs, and also the fraction of the novice CAMs found within the consensus-of-two CAMs across different pixel thresholds. The consensus-of-two CAMs were a smaller and more focused subset of the novice CAMs (Fig. 5d).

### Ensemble learners bolstered performance and were robust to intentionally noisy “annotators”

**Fig. 6:**
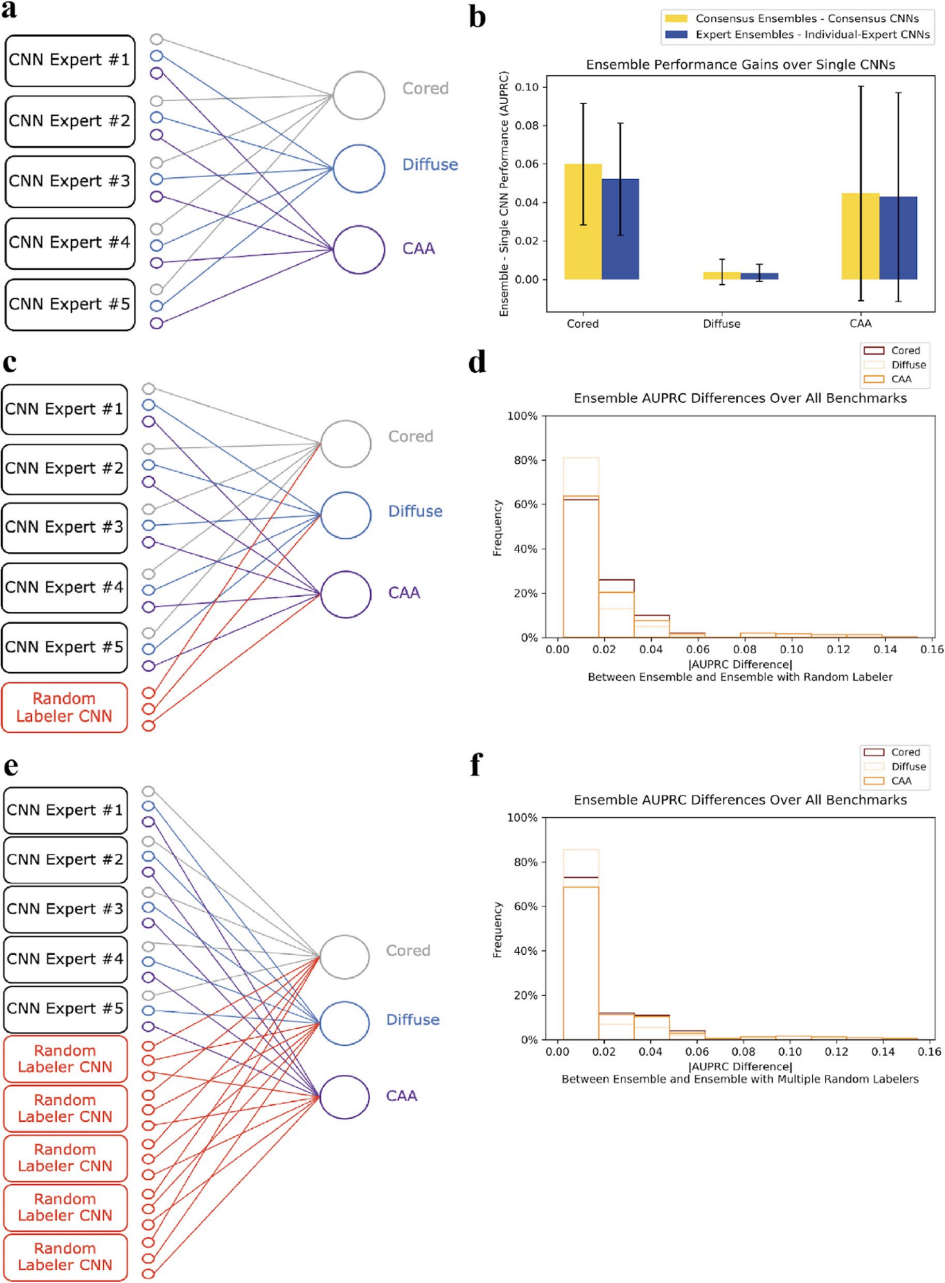
Ensembles improve performance and are robust to false information. **a** Five trained individual-expert CNNs, combined by a trainable sparse affine layer, make up an ensemble model. The training process simply determines how to best weigh and combine each CNN’s existing class predictions. **b** Ensembling on average increases performance for each Aβ class, and for both consensus and individual benchmarks. Performance gains are calculated by averaging each ensemble’s AUPRC on the hold-out test set minus the corresponding individual-expert CNN’s AUPRC on the same set, across all ten benchmarks (Methods). **c** We tested ensembling with a random labeler CNN, trained using a randomly shuffled permutation of labels with the same class distribution ratios as the five expert annotations. **d** Ensemble performance is largely unaffected by inclusion of a random labeler CNN. Density histogram of AUPRC performance differences for each Aβ class between the normal ensemble and the ensemble with a single random labeler CNN. Each ensemble is evaluated on all ten benchmarks (five individual-expert benchmarks, five consensus benchmarks), and the absolute value of the performance differential (x-axis) is calculated and binned for each class. **e** Ensemble architecture with multiple random labeler CNNs, each trained on a different permutation of randomly shuffled labels. **f** Ensemble performance is largely unaffected by inclusion of five random labeler CNNs. Same density histogram as in **d**, but comparison is between normal ensemble and ensemble with five random labeler CNNs injected

We further tested multi-rater strategies by creating an ensemble of individual-expert models (no novices) and taking a “wisdom of crowds" approach [42]. One approach would be to simply take the predictions of the different expert models and use a majority-voting scheme. However, weighting each model equally would ignore the specific and differing expertise the models gleaned during training. Other approaches assign differently weighted votes to different models [25, 43]. Accordingly, we combined the individual-expert models in a learnable way, such that the resulting ensemble learned how to properly weight each contributing network’s Aβ class predictions to maximize overall performance. We theorized that the ensemble would learn how to combine the strengths of individual-expert models. The ensemble training did not modify the individual CNNs, but rather learned how to weight each constituent network to maximize performance for a given annotation benchmark (Fig. 6a). We created ensembles for each of the phase-one annotation sets: both individual-expert annotation sets, as well as consensus annotation sets (“Methods” section).

This ensemble approach bolstered performance, and improved over individual-expert CNNs in identifying CAAs. On average, ensembles outperformed single (non-ensembled) CNNs (Fig. 6b). Performance differences for diffuse class detection were relatively unaffected, likely because these examples were already highly prevalent. Rather, we observed performance gains for the less prevalent but pathologically important minority classes (cored and CAA). For consensus ensemble cases, in which we trained different ensembles to reproduce consensus-of-*n* annotations from *n* = 1 to *n* = 5, the ensembles matched or slightly outperformed single CNNs trained to mirror the consensus.

Next, we investigated whether these performance gains were robust to having a poor annotator included in the ensembles (Fig. 6c). We modeled this by creating a random labeler, who assigned randomly shuffled annotations that matched the average class distribution of all experts. When we included a random annotator CNN in the ensembles, the ensembles successfully learned to reject the noisy information from the random labeler. Performance was largely unaffected by the random annotator CNN, with differences in performance averaging to less than 0.01 in AUPRC across every permutation of ensemble model and evaluation benchmark (Fig. 6d).

In a separate experiment, we also included five independent random labeler CNNs in the ensembles (Fig. 6e), and we observed the same resilience and robustness. The ensembles successfully learned to ignore noisy contributors, and maintained performance even at a population that consisted of 50% poor labelers who inserted random information (Fig. 6f). Hence, we found ensembles resulted in better performance, and were robust to multiple poor annotators at little perceived risk of random information. By contrast, including random annotations into the consensus-of-*n* schemes would proportionally dilute the training data signal.

### Annotators were self-consistent during phase-two

**Fig. 7:**
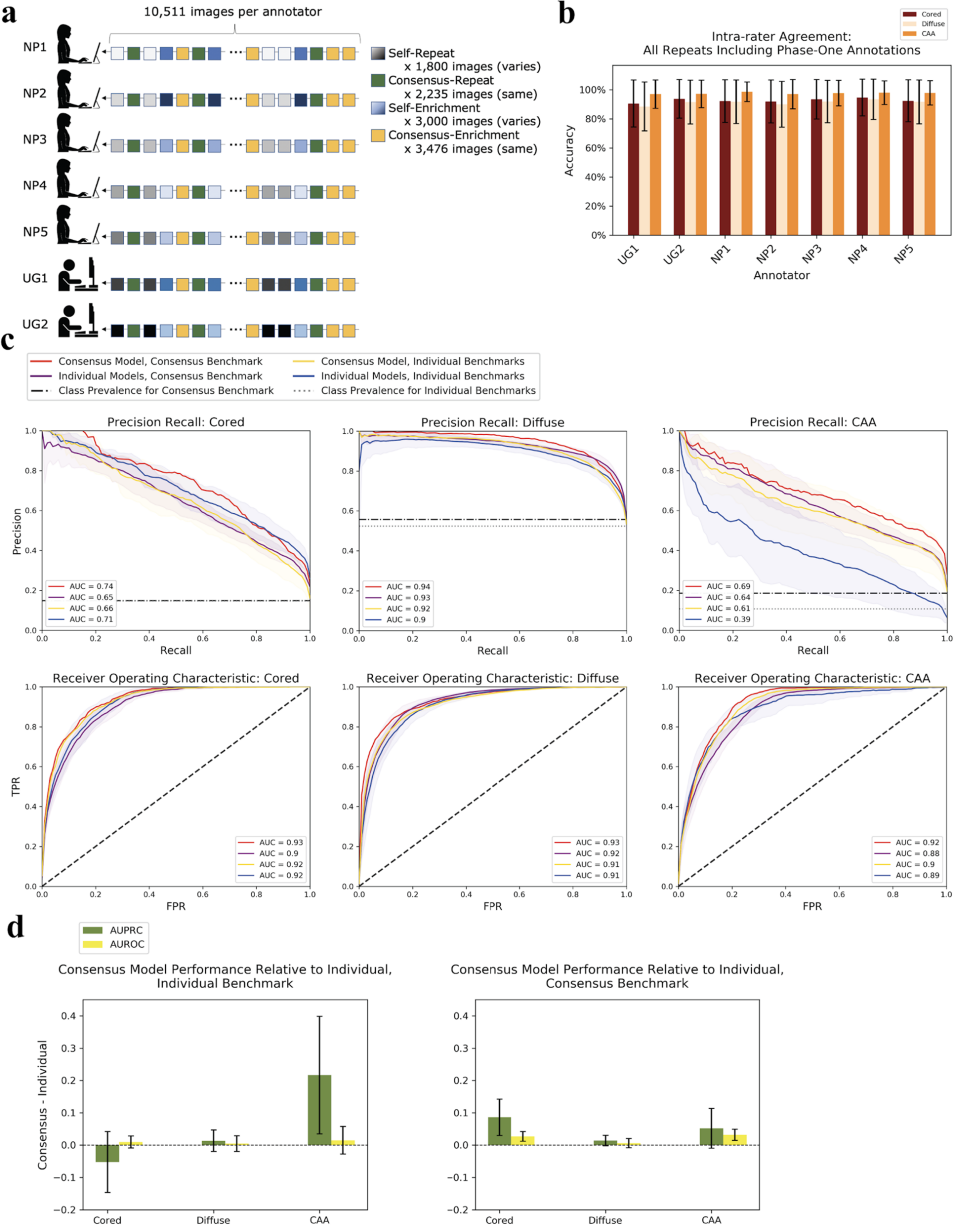
Models prospectively predict human annotation, with consensus models performing the most consistently. **a** Schematic of the phase-two annotation protocol. These images fall under one of four categories: self-repeat, consensus-repeat, self-enrichment, and consensus-enrichment. See Methods for a detailed description of these categories. Each annotator is given the same order of image categories. Gradients of different colors indicate images from the same category. These gradients are depicted to reinforce the fact that each annotator received a different set of images for the self-repeat and self-enrichment categories. **b** Intra-rater agreement is measured as the accuracy at which each rater consistently annotates repeats of the same image (both self-repeat and consensus-repeat). We include image labels from phase-one in this intra-rater calculation. The x-axis indicates the annotator, and the y-axis indicates intra-rater accuracy. Accuracies are averaged over each set of repeated images. Novices achieved an average intra-rater agreement accuracy of 0.92 for cored, 0.90 for diffuse, and 0.97 for CAA. Experts achieved an average intra-rater agreement accuracy of 0.93 for cored, 0.92 for diffuse, and 0.98 for CAA. **c** Precision recall plots and receiver operating characteristic (ROC) plots for the consensus model versus the individual-expert models. Two different benchmarks are used—truth according to the individual annotators, and truth according to a consensus-of-two scheme. The shaded regions indicate one standard deviation in each direction centered at the mean. The consensus model evaluated under a consensus benchmark (red line) has no variation by definition. **d** Summarizes panel (**c**). Bar graphs depict the average performance of the consensus model minus the average performance of the individual-expert models (y-axis). Individual benchmark for figure left, consensus benchmark for figure right. Error bars show one standard deviation centered at the mean

We conducted phase-two annotations six months after the completion of phase-one, using the same web platform as phase-one (Fig. 7a, Methods). Encouragingly, each annotator achieved high intra-rater agreement for all of the repeat images, and was able to consistently annotate and reproduce the same labels across the six-month gap (Fig. 7b). Each expert achieved greater than 90% intra-rater accuracy among all Aβ classes, while novices achieved greater than 88% accuracy. There was no significant difference in intra-rater agreement between self-repeats and consensus-repeats (Additional file 1: Figure S10).

### Models prospectively enriched for minority Aβ classes and favored consensus learning

Before completing this phase of the study, it was not clear whether a model trained on an individual annotator would prospectively perform the filtering and prediction task the best, as compared to a model trained from expert consensus. Consequently, from the 10,511 phase-two annotations across five expert annotators (i.e. total of 52,555 phase-two expert annotations), we created performance benchmarks to compare strategies. Models were assessed by either taking the individual expert’s labels as truth (called the “individual benchmarks,” five independent benchmarks), or by taking a consensus-of-two experts as truth (called the “consensus benchmark”, one benchmark). We found that regardless of the selected benchmark, the consensus model performed better than individual-expert models in most cases, despite the fact that the individual-expert models were trained specifically under the same human annotators that provided the individual benchmarks (Fig. 7c).

Both types of models were able to perform well prospectively for each Aβ class, with the consensus model outperforming individual-expert models on average in AUPRC and AUROC (by as much as 0.22 ± 0.18 for CAA AUPRC, Fig. 7c, d). For all classes and for all metrics, the consensus model evaluated on the consensus benchmark performed best. Furthermore, evaluating under a consensus benchmark as opposed to an individual benchmark resulted in either the same or better performance across models and across success metrics. Taken together, the consensus model was robust to differing annotations of five different experts. On average, this model exceeded performance of individual-expert models even on their own benchmarks that they were trained to mimic, and also on the consensus benchmark (Fig. 7d).

These results held for each class and each metric, with the sole exception being the cored AUPRC. In this case, individual-expert models slightly outperformed the consensus model under the individual benchmark (but not for the consensus benchmark, under which the consensus model was superior). This was consistent with the phase-one observation that cored-plaque labeling had more idiosyncrasies (Fig. 1c). Hence, we expected individual-expert models to be better performers on individual benchmarks for this class. Furthermore, evaluating individual-expert models under a consensus benchmark did not improve performance over the individual benchmark, likely for the same reason.

## Discussion

We developed a scalable, objective, and consistent way to automate and compile annotations of multiple neuropathology experts. Four points merit emphasis: (1) We automated individual and consensus annotations across datasets from five experts and three staining procedures from different institutions, advocating for generalizability; (2) When we prospectively validated these models in a research setting, a consensus model was robust under different benchmarks and provided a high-performance approach to automatic and standardized labeling; (3) The models were interpretable and showed increased pixel specificity with increased expertise; and (4) Ensemble models generally performed better and were robust to intentionally randomized information. We note however that the experimental task differed from the daily practice of neuropathological annotation, where experts would commonly assess these objects using varying magnifications and localizations.

DL requires annotated datasets that define how to train a model and quantify its success. However, the classification of neuropathologies continues to evolve, with different experts having different focus areas. Consequently, two competing hypotheses regarding the best annotation strategy might be reasonable. In the first, a DL model trained on an individual might best leverage that single expert’s unique intuition and decision-making procedures for edge cases. Conversely, in a second strategy, a consensus approach to annotations might instead leverage a wisdom-of-the-crowd logic to remove individual variance in a consolidated consensus model, as long as there is common underlying signal across the cohort. Depending on the consensus strategy, such a consolidated model could balance between strict-but-conservative expert consensus (the logical intersection) versus an inclusive but potentially overly-permissive consensus (the logical union).

In this study, we found that a balanced consensus-of-two strategy slightly favoring permissiveness performed better in nearly every model-training and model-benchmarking scenario (Fig. 4c). This was striking, as the consensus-trained model outperformed the individual-expert models even when unfairly assessed against those self-same individual-expert benchmarks (Fig. 4). One might reasonably have otherwise expected individual-expert models to better encode their particular expert’s pathological training and intuition, but this was generally not the case. We conclude from this observation that neuropathological labeling across thousands of independent annotations was sufficiently consistent across the cohort that a permissive consensus model computationally codified commonly-held expertise in neuropathological identification—despite noticeable differences in individual annotations (Fig. 2). We are unaware of any previously published work that operated on a cohort of independent expert annotations at this scale. Whereas other DL pathology applications rely on the independent assessments of individual pathologists [45, 25, 46, 47, 22, 48], we believe this is the first study to demonstrate that learning expert consensus within pathology provides robust and superior performance over learning individual assessments.

Encapsulating a consensus strategy into a model may be useful when an expert cohort or adequate labeling time are unavailable. In such cases, having an automated algorithm to provide consistent diagnoses from a wisdom of crowds would be beneficial. Hence, phase-two provided a proof-of-concept to prospectively assess whether these models could serve as a practical co-pilot and enrich for rarer but important neuropathologies within an unexplored dataset of n = 275,879 candidate neuropathologies from new patients (Fig. 7a). This represented a clinical scenario in which the pathologist wishes to rapidly curate sparse pathology examples, or to have a DL model act as an individualized assistant enriching for important but infrequent morphologies, which may improve understanding of these phenotypes and provide opportunity for overcoming sparsity challenges.

In the same vein of utilizing models to improve pathological understanding, the models learned directly from the image data and became more focused in their feature detection as experience of the labeler increased from novice to expert consensus (Fig. 5a). These results suggest potential for application in the training of new pathologists, using models to identify critical image features and thereby visually illustrate for new trainees the phenotypes relevant for labeling. A future direction is to explore this new opportunity of DL facilitating human learning, as opposed to the more common framework of humans facilitating DL, ultimately leading to a hybrid-feedback loop through active learning [49].

The ensemble models may provide an opportunity of weighting individualized model input to tailor a specific annotator’s expertise to specific tasks or cases. Subsequent to a prediction, we can inspect the interplay of contributor-specific weights (Additional file 1: Figure S11). In this way, individualized contributions encoding complementary and situation-specific expertise do not get overwhelmed as they might otherwise under a simple vote averaging. Although we do not expect labelers to intentionally introduce false information, the ensembles’ robustness to unreliable annotations encourages assembling numerous and diverse labelers to facilitate accurate and learnable labeling.

Several limitations to this study inform its practical adoption. Researchers may not have access to necessary computational resources or training data for DL. However, cloud computing resources are becoming more accessible, and open-data sharing is also possible through data-hosting services. We are openly releasing this study’s annotated datasets and trained models (https://github.com/keiserlab/consensus-learning-paper). Turning to limitations in interpretability methods, we note that guided grad-CAM devolves to being a visual edge-detector in some settings [50]. However, this was not the case in our study because saliency maps calculated on the same input image but different Aβ class substantially differed (Additional file 1: Figure S12). Nonetheless, a future direction would be exploring a range of independent saliency mapping techniques [51–53]. Finally, prospective effectiveness outside this cohort remains open to exploration. Whereas this study was consistent with our earlier work leveraging one expert annotator [22] and its independent application at another institution [23], we expect larger cohorts and institutionally-diverse datasets (including persons from multiple socioeconomic backgrounds) with different neuroanatomic areas and staining techniques will facilitate more comprehensive standards in neuropathology. For instance, performance for CAA with 6E10 was lower than for other stains (Additional file 1: Figure S5). This could be due to its smaller representation in the test set (Additional file 1: Figure S13). Although this study’s cohort of five experts was institutionally diverse, it could be improved by capturing greater variability from the broader community. Despite these limitations, the models accurately learned the annotation practices of five experts and of their shared expertise, indicating the method’s generalizability.

We imagine this neuropathology study might apply also to anatomic pathology and other areas of medical research. Any medical discipline that leverages human expertise could benefit from taking advantage of expert diversity and consensus to make automated and consistent diagnoses. Models and annotated datasets developed and shared across the community could be progressively refined as ever-improving metrics and deployable tools of shared ground truth. Whereas we focused on automatic image annotation, the concept may generalize to other domains and data types where expert annotation is crucial. Although a consensus-of-two experts was best in this particular study, this may not hold for other studies or for a different cohort or pathology, and indeed improved ensemble modeling techniques may be the strongest approach (Fig. 6). However, the resilience and robustness of the consensus model indicated that training from a consensus was better than relying on individual assessments, despite high expert intra-rater reliability even over a half-year gap (Fig. 7b). We hope that developing and openly sharing consistent, accurate, and automated DL methods and their datasets can facilitate standardization and accelerate quantitative pathology as a freely available community resource. These results point to a means to continually refine rapid and reliable models to identify amyloid neuropathologies, derived from the consensus expertise of an expanding neuropathologist cohort.

## Conclusion

Whereas deep learning models can learn from a single neuropathology expert [22], we wondered whether models learning from an expert cohort would find common ground. Would consensus and ensemble models leverage the strengths of complementary expertises, or instead founder on different assessments? This question would clearly have a crucial impact on the increasing integration of deep learning with the practice of neuropathology, however, to our knowledge, no dataset existed to address these questions. Thus we collected a dataset of 150,000 expert-annotated amyloid neuropathologies. A volunteer cohort of five neuropathology experts from institutions across the country and world each independently annotated 30,000 potential amyloid neuropathologies. Using this dataset, we assess inter- and intra-rater reliability at scale. We demonstrate that deep learning algorithms trained from multiple experts via a consensus strategy is a robust, effective, and interpretable means to label pathologies that may have expert disagreement. Shareable and improvable common-ground tools are imperative for standardizing quantitative pathology. We show that this method is effective on an institutionally and methodically diverse dataset, as a first test of its generalizability to other pathological sites and tasks. Moreover, through ensembling, we show the contribution of larger community involvement increases performance with little risk from confounding annotations. Performing a prospective study during phase-two, we also show that consensus learning can be used in a real-world clinical research context to successfully enrich rarer pathologies. For broad impact and to put these tools into expert community hands, we release the entire annotated dataset and open-source software to freely use as both a labeling tool and a resource for future research.

## Supplementary Information

**Additional file 1**. Contains all of the supplementary figures and corresponding legends.

**Additional file 2**. Contains the meta data for all WSIs.

## Data Availability

The raw WSIs, the color-normalized 1536 × 1536 pixel tiles, and the final 256 × 256 pixel images and their annotations are all freely available, and can be found at: https://osf.io/xh2jd/. All code and models are freely available at: https://github.com/keiserlab/consensus-learning-paper

## Abbreviations

DL: Deep learning
CAA: Cerebral amyloid angiopathy
Aβ: Amyloid beta
WSIs: Whole slide images
IRB: Institutional Review Board
HSV: Hue saturation value
CNN: Convolutional neural network
NP#: Neuropathologist #
UG#: Undergraduate novice #
AUROC: Area under the receiver operating characteristic
AUPRC: Area under the precision recall curve
CAMs: Class activation maps
